# Measurement of shear stress-mediated intracellular calcium dynamics in human dermal lymphatic endothelial cells

**DOI:** 10.1152/ajpheart.00744.2014

**Published:** 2015-01-23

**Authors:** M. Jafarnejad, W. E. Cromer, R. R. Kaunas, S. L. Zhang, D. C. Zawieja, J. E. Moore

**Affiliations:** ^1^Department of Bioengineering, Imperial College, London, England;; ^2^Department of Medical Physiology, Texas A&M Health Science Center, Temple, Texas; and; ^3^Department of Biomedical Engineering, Texas A&M University, College Station, Texas

**Keywords:** shear stress, intracellular calcium, lymphatic endothelial cell

## Abstract

The shear stress applied to lymphatic endothelial cells (LEC) by lymph flow changes dramatically under normal conditions as well as in response to disease conditions and immune reactions. In general, LEC are known to regulate the contraction frequency and strength of lymphatic pumping in response to shear stress. Intracellular calcium concentration ([Ca^2+^]_i_) is an important factor that regulates lymphatic contraction characteristics. In this study, we measured changes in the [Ca^2+^]_i_ under different shear stress levels and determined the source of this calcium signal. Briefly, human dermal LEC were cultured in custom-made microchannels for 3 days before loading with 2 µM fura-2 AM, a ratiometric calcium dye to measure [Ca^2+^]_i_. Step changes in shear stress resulted in a rapid increase in [Ca^2+^]_i_ followed by a gradual return to the basal level and sometimes below the initial baseline (45.2 ± 2.2 nM). The [Ca^2+^]_i_ reached a peak at 126.2 ± 5.6 nM for 10 dyn/cm^2^ stimulus, whereas the peak was only 71.8 ± 5.4 nM for 1 dyn/cm^2^ stimulus, indicating that the calcium signal depends on the magnitude of shear stress. Removal of the extracellular calcium from the buffer or pharmocological blockade of calcium release-activated calcium (CRAC) channels significantly reduced the peak [Ca^2+^]_i_, demonstrating a role of extracellular calcium entry. Inhibition of endoplasmic reticulum (ER) calcium pumps showed the importance of intracellular calcium stores in the initiation of this signal. In conclusion, we demonstrated that the shear-mediated calcium signal is dependent on the magnitude of the shear and involves ER store calcium release and extracellular calcium entry.

the lymphatic system plays important roles in fluid and protein balance, lipid absorption, and immune response in the body. Any dysfunction of this system results in tissue accumulation of lymph, or lymphedema (40), as well as the induction of inflammatory responses (23, 39, 50). The biology of the lymphatic system has been the subject of several review articles in the past decade (32, 45, 56, 57). Some of these have recognized the importance of the unique mechanical environment of the lymphatic vasculature, but little is known about the mechanoregulatory mechanisms of this system. In particular, lymphatic pumping appears to rely on specific mechanical cues (e.g., flow and pressure) to modulate pumping activity (33). Upon a modest increase in transmural pressure (stretch) in isolated rat mesenteric lymphatic vessels, the vessel tone changes, the phasic contraction strength of the lymph pump increases, and the frequency of the pumping is elevated (19, 33). On the other hand, when a flow (shear) is induced in these vessels, the contraction frequency drops threefold supporting the importance of mechanotransduction in regulation of pumping activity (26, 33). Because lymphatic endothelial cells (LEC) are subjected to both flow-induced shear stress and stretching as the vessel contracts and relaxes, these cells represent a logical target for investigating important mechanosensitive control mechanisms. Although the importance of mechanical shear stress has been investigated extensively in blood endothelial cells (EC) (2, 13, 21, 30, 42, 46, 47), very few studies have attempted to unveil the effect of mechanical forces on LEC both ex vivo and in vitro (16, 24).

In vascular EC, shear stress regulates the production of vasoactive substances such as nitric oxide (NO) (3, 27), endothelin-1 (12, 28, 49), and prostacyclin (8) as well as intracellular ions, the most important of them being calcium (17, 21, 42). Intracellular calcium is a ubiquitous key second messenger that can regulate NO synthesis, cytoskeleton reorganization, and endothelial permeability. Rapid increases in intracellular calcium lead to activation by phosphorylation of endothelial nitric oxide synthase (eNOS) resulting in subsequent release of NO, the most important vasodilator in the body (11, 15, 27). Furthermore, the Ca^2+^-calmodulin complex activates myosin light chain kinase (MLCK) leading to increased cell contractility/tone and cytoskeletal reorganization that modulates monolayer permeability (48). Although the physiological and pathological effects of vasodilation and permeability changes are well documented in blood vessels, there is still a paucity of published works about their role in lymphatic vessels. In particular, permeability changes can potentially increase the exchange of important immunomodulatory signals including antigens and cytokines between the lymphatic vessels and surrounding tissue, thus potentially facilitating the mobilization of dendritic cells (34).

EC calcium dynamics can be regulated by agonists (e.g., bradykinin, acetylcholine, etc.) and mechanical forces such as shear stress (17, 21, 31). Agonists bind to G protein-coupled receptors (GPCR) or receptor tyrosine kinases (RTK), thereby activating phospholipase C (PLC) and cleavage of phosphatidylinositol bisphosphate (PIP2) resulting in formation of inositol (1,4,5)-triphosphate (IP_3_) (4, 6, 51). IP_3_ then binds to the IP_3_ receptor (IP_3_R) on the endoplasmic reticulum (ER) membrane and releases the intracellular calcium stores into the cytosol. Similarly, experiments have also shown a transient upregulation of IP_3_ in EC at the onset of shear stress (7, 37). This suggests shear stress can utilize parts of the same pathway as receptor-operated mechanisms to mobilize the ER calcium.

Although several mechanosensors of shear stress in EC have been proposed, such as the glycocalyx (46), plasma membrane channels (13), matrix adhesion proteins (52), primary cilia (35), and membrane receptors (e.g., GPCRs and RTKs) (20), the contributions of each of these mechanosensors in the final signal remain to be elucidated (1). For shear stress-induced intracellular calcium signaling, ATP-binding purinergic membrane receptors have been demonstrated to play an important role (17, 22, 55). However, the pathway by which intracellular calcium is regulated under low concentrations or absence of ATP is incompletely resolved (17, 42).

With the growing understanding of the importance of mechanics and mechanotransduction in the lymphatic system and the acknowledged high sensitivity of lymphatic tissues to pressure and flow, a dynamic measurement of intracellular calcium is crucial for a better understanding of the regulatory mechanisms at the cellular scale, which can then be integrated into models to help explain the responses seen experimentally at the tissue and organ levels. The aims of this study were *1*) to measure intracellular calcium dynamics in lymphatic EC under different levels of shear stress; and *2*) to identify the source of the signal by manipulating both intracellular stores and extracellular calcium concentration.

## METHODS

### 

#### Cell culture.

**Fig. 1.**
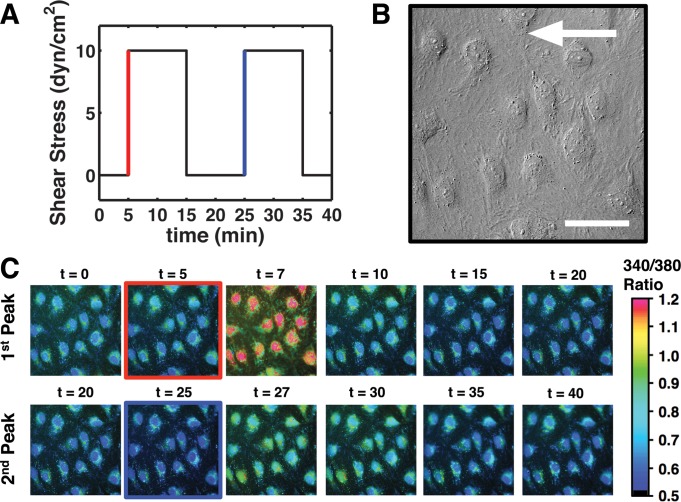
Intracellular calcium concentration ([Ca^2+^]_i_) increased under step changes in shear stress. *A*: cells were kept under no-flow condition under the microscope for 5 min before a step change in flow increased shear stress to 1, 3, or 10 dyn/cm^2^ (the red line). The shear stress was kept constant in the elevated level for 10 min after which it was dropped back to zero. 10 min after the shear stress stopped, the same pattern of on-off in shear stress was repeated (starting with the blue line). *B*: differential interference contrast (DIC) images were captured at each time-point to visualize the state of human dermal lymphatic endothelial cells (HDLEC) in the flow chambers. The white arrow shows the direction of the flow. Scale bar = 50 μm. *C*: pseudocolor 340/380 ratio images show a stable [Ca^2+^]_i_ before the flow started. After a step increase in shear stress (red-bordered image), the signal peaked in a few minutes (at ∼*t* = 7 min) and then immediately started to decline to reach levels near the baseline. Ten minutes of no-shear condition resulted in a stable baseline followed by a 2nd increase in shear stress (blue-bordered image). The [Ca^2+^]_i_ signal peaked again (at ∼ t = 27 min), but the [Ca^2+^]_i_ levels were not as high as the initial response in this example. This example is for shear steps of 10 dyn/cm^2^ done on the same cells in the DIC image of *B*. Red and blue lines and borders in *A* and *C* correspond to the same events.

A commonly used commercial cell-line of juvenile human dermal lymphatic endothelial cells (HDLEC) was obtained from PromoCell. The exact lymphatic vascular origin of the HDLEC is not precisely known since the dermal lymphatic network contains lymphatic capillaries and precollector and collector lymphatics. HDLEC were cultivated in microvascular endothelial growth medium 2 (MV2) from PromoCell. The media of the flasks were changed three times a week, and HDLEC with passages less than six were used for all the experiments. Due to the origin of the HDLEC from human foreskin, two batches of these cells were required to perform this study using only low passage cells. Flow chambers were coated with 2% gelatin for 4 h at 37°C and then were seeded with the HDLEC at 50% confluence. The cells adhered to the gelatin coating on the coverslips at the bottom of the chambers. HDLEC were cultivated under static conditions in the chambers for at least 3 days or when more than 90% confluent (Fig. 1*B*). The culture media were replenished every 24 h after seeding the cells.

#### Shear stress apparatus.

Polydimethylsiloxane (PDMS) single-use microfluidic channels were formed to apply controlled shear stress on EC. Standard photolithography was used to fabricate the master molds and the PDMS chambers were replicated from the mold using soft-lithography (43). Briefly, 100- to 120-μm film of SU-8 2050 negative photoresist was coated on 3-in. silicon wafers. After baking steps at 65 and 95°C, the film was exposed to ultraviolet light through a dark-field mask to transfer the design to the film. At the end, the patterns were developed in Microchem SU-8 developer reagent. The replicated PDMS parts were punched, autoclaved, dried, and then bound to glass coverslips (#1) ready for cell culture (43). A computer-controlled syringe pump was used to apply step changes in shear stress on the cell monolayer in the chambers. The baseline signal was acquired for the first 5 min, and then flow was started to obtain a constant shear stress (1, 3, or 10 dyn/cm^2^) for 10 min. Flow was then paused for 10 min for restabilization before applying a second onset and offset of the shear stress (Fig. 1*A*). Although the physiologic waveform of shear stress on lymphatic vessel wall is more dynamic and complicated, this simplified waveform facilitated investigation of the basic response of HDLEC to constant shear stress levels within the physiological ranges we have previously observed in small collecting lymphatics (16). Shear stress was determined using the relationship between flow rate and shear stress between the two parallel plates, namely τ_*w*_ = 6μ*Q*/*h*^2^*w* where τ_*w*_ is shear stress, μ is viscosity of media, *Q* is flow rate, *h* is the channel height, and *w* is the channel width.

#### Calcium measurement.

HDLEC were serum starved in DMEM/F-12 (GIBCO) for at least 2 h before the experiments started. Cells were then incubated with 2 μg/ml fura-2 AM in F-12 media containing 0.1% DMSO for 30–45 min. Then, cells were washed with F-12 or physiologic saline solution (PSS: 145 mM NaCl, 4.7 mM KCl, 2 mM CaCl_2_, 1.17 mM MgSO_4_, 1.2 mM NaH_2_PO_4_, 5 mM dextrose, 2 mM sodium pyruvate, 20 nM EDTA, 3 mM MOPS, and 2% HI FBS) and incubated for another 30 min for deesterification. The Ca^2+^-free PSS was prepared with similar formulation as PSS but contained 3 mM EDTA instead of CaCl_2_. Finally, chambers were transferred to the flow setup, and experiments were run using the desired buffer (F-12, PSS, or Ca^2+^-free PSS).

Pairs of fluorescent images were taken by exposure to 340- and 380-nm excitation wavelengths. The image pairs were obtained every 15 s using an IX81 microscope (Olympus)-based system as described previously (58). Differential interference contrast images were also captured in each time-point to monitor cell morphology. Multiple regions of interest (∼20–40 ROIs), each containing a single EC, were selected for data analysis in each experiment. At each time point, the background fluorescent signal in each wavelength was measured in a region not containing any cells and was subtracted from all the ROIs for the respective wavelength. Dividing background-subtracted 340-nm images by 380-nm images resulted in ratio images (Fig. 1*C*). The mean ratio was first calculated in each ROI and then averaged between all ROIs within a single chamber to obtain a trace for mean changes in ratio for each experiment. Furthermore, the mean data from several experiments (*n* = 4–9) were averaged to calculate the mean ratio for each type of experiment. Additionally, SE was calculated using the mean values from each condition to represent variability between individual experiments and not variability of the response within each experiment.

The ratio of 340-nm images over 380-nm images (340/380 ratio) can be converted to spatial calcium concentration field using a calibration equation in the form of

$${\left[{\text{Ca}}^{2+}\right]}_{\text{i}}=K_{\text{d}}\frac{\left[R-R_{\min}\right]}{\left[R_{\max}-R\right]}\frac{F_{\max}^{380}}{F_{\min}^{380}},$$

where [Ca^2+^]_i_ is intracellular calcium concentration, *K*_d_ is dissociation constant for fura-2-calcium binding, *R*_min_ and *R*_max_ are minimum and maximum ratios measured under saturating levels and absence of calcium, respectively, and *F*_min_^380^ and *F*_max_^380^ are fluorescence intensity with 380-nm excitation measured under saturating levels and absence of calcium respectively.

#### Curve fits.

MATLAB (R2013a) curve-fit toolbox was used to fit a linear function to the upstroke and an exponential function to the downstroke of each calcium signal. For the upstroke, linear least-squares method was used to fit a linear function in the form of ratio = *a*_1_ × *t* + *a*_2_ to the data, where *a*_1_ represents the slope of the upstroke linear fit. This parameter was calculated for the averaged signal of each type of experiment for the first and second peaks. As for the downstroke, nonlinear least-squares method with Trust-Region algorithm was used to fit an exponential function in the form of ratio = *b*_1_ × exp[(*t* − *t*_0_)/*b*_2_] + *b*_3_ to the averaged data for each type of experiment. The minimum of the signal in the interval from the peak to 5 min after the flow stop is used as the *b*_3_ parameter for each type of experiment, while *t*_0_ is the time that the signal started to decrease and *b*_2_ parameter represents the time constant by which the signal drops.

#### Statistics.

Data are generally reported as the means ± SE. ANOVA was used to compare the result of each peak with its control and also to compare the two peaks in the same type of experiment. Wherever more than two groups were compared, Bonferroni correction was used to ensure statistical significance between group means. All the statistical analyses were done in MATLAB R2013a with a critical *P* value of 0.05.

## RESULTS

**Fig. 2.**
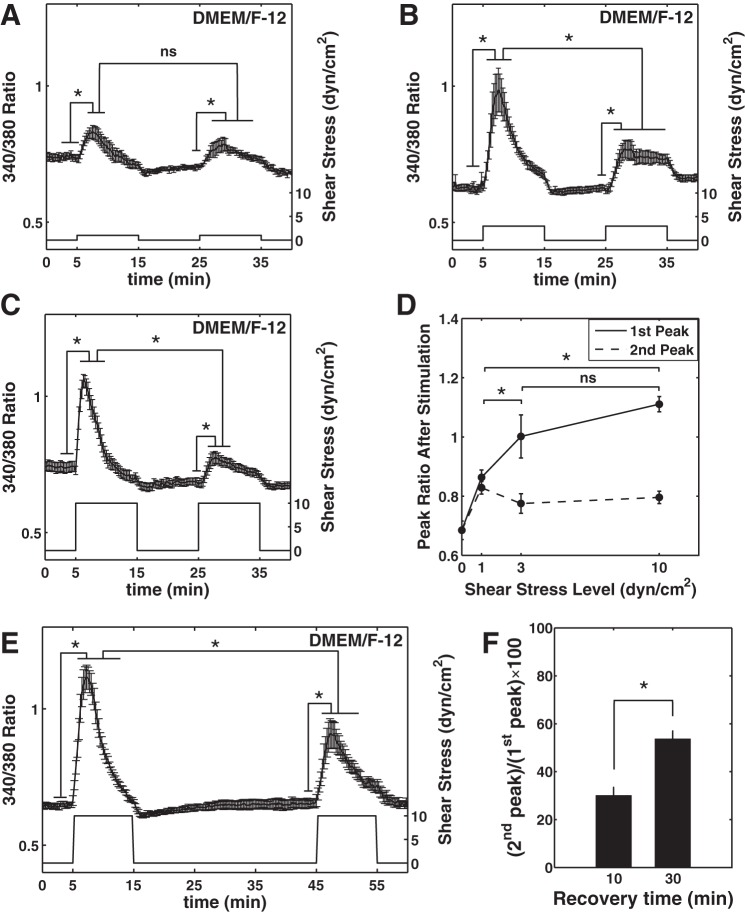
[Ca^2+^]_i_ dynamics under different levels of shear stress. *A–C*: shear stimuli of 1 (*A*), 3 (*B*), or 10 (*C*) dyn/cm^2^ showed significant increase in averaged ratio measurements for both shear stimuli compared with respective baselines. However, only for shears higher than 1 dyn/cm^2^ was the 2nd peak significantly smaller than the 1st peak. The ratio signal is the trace with error bars on the primary axis and the shear stress is the square wave shown on the secondary axis. The measurements are reported as means ± SE (*A*: *n* = 9; *B*: *n* = 4; and *C*: *n* = 9). *D*: average peak response to the 1st shear stimulus shows that the 3 dyn/cm^2^ response is not significantly different from the 10 dyn/cm^2^ response meaning that [Ca^2+^]_i_ response plateaus at ∼3 dyn/cm^2^. The peaks for *D* are calculated in each experiment individually and then are averaged over the number of experiments. *E*. When the no-shear period (recovery time) between the 2 stimuli was increased from 10 to 30 min, the 2nd peak significantly increased (means ± SE; *n* = 6). *F*: with 10-min recovery time, the 2nd peak height was only 30% of the initial peak height, however, when this time was increase to 30 min, the 2nd peak height significantly increase to 54% of the initial peak height (*P < 0.05; ns: nonsignificant).

A step change in shear stress (10-min long) applied to the HDLEC resulted in an increase in [Ca^2+^]_i_ that peaked and then generally decayed back towards basal levels (Fig. 2, *A–C*). The magnitudes of the peak rise and the decay were dependent on the shear stress applied. For example, upon initiating a shear stress of 10 dyn/cm^2^, the fluorescence ratio (the index of [Ca^2+^]_i_) increased significantly (Fig. 2*C*), within 1.75 min (on average for calcium-containing DMEM/F12) from a basal ratio of 0.74 ± 0.02 to a peak value of 1.11 ± 0.03. The calcium signal then decreased exponentially even though shear stress remained elevated (Fig. 2*C*). Indeed, at the end of the 10 min of constant shear (10 dyn/cm^2^), calcium had fallen to values below the original baseline. Shear stress was then returned back to zero for 10 min before a second step of similar shear stress was applied to evaluate the recovery capability of these cells. During the 10-min “resting” period when shear stress was back to zero, the calcium signal generally recovered back toward the initial baseline, often falling below the initial basal level (Fig. 2, *A–C*). When a second identical shear stimulus was applied after the resting period (Fig. 2*C*), the magnitude of the second peak (0.79 ± 0.02) was significantly lower than the first. Based on the calibration constants, a ratio of 1.11 ± 0.03 for the first peak indicates 126.2 ± 5.8 nM, and a ratio of 0.79 ± 0.02 for the second peak indicates 57.3 ± 4.5 nM.

The [Ca^2+^]_i_ transients were shear-magnitude dependent for the first peak while the magnitude of the second peak was not significantly dependent on the magnitude of the applied shear and was significantly lower than the first peak for shears higher than 1 dyn/cm^2^ (Fig. 2, *A–D*). The initial peaks of [Ca^2+^]_i_ measured under shear stresses of 1, 3, and 10 dyn/cm^2^, elicited peak ratios of 0.86 ± 0.03, 1.00 ± 0.07, and 1.11 ± 0.03, respectively (Fig. 2, *A–D*) while the time to reach the peak was 3.3 ± 0.1, 3.0 ± 0.2, and 1.8 ± 0.1 min, respectively. For the second stimulus of the same shear stress magnitudes, the peak calcium ratios were 0.82 ± 0.02, 0.77 ± 0.03, and 0.79 ± 0.02 with peak times of 4.7 ± 0.3, 3.6 ± 0.1, and 3.1 ± 0.1 min, respectively (Fig. 2, *A–D*).

We hypothesized that a longer resting time between two stimuli would increase the restoration of the ER calcium stores, thereby resulting in greater recovery of the second peak. With application of 10 dyn/cm^2^ shear, a 10-min no-shear period resulted in a second peak ∼30% of the height of the first peak, whereas when this recovery time was increased to 30 min, the second peak height increased to ∼54% of the first peak height (Fig. 2, *E* and *F*). In both cases, the second peak was significantly smaller than the first peak and the 30-min recovery peak was significantly higher than the 10-min recovery peak (Fig. 2*F*). This change in the size of the second peak did not affect the time at which the signal reached the peak value (3.1 ± 0.2 min).

**Fig. 3.**
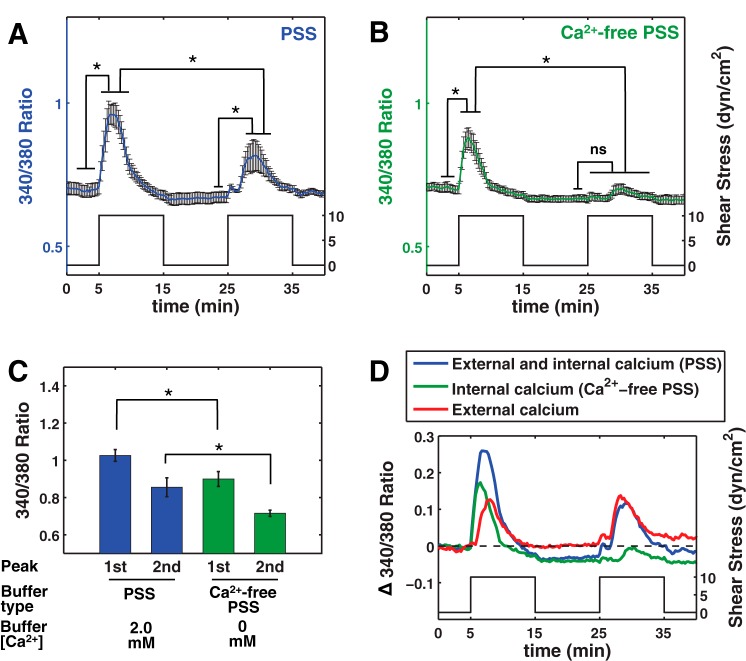
Effect of extracellular calcium concentration on [Ca^2+^]_i_ dynamics. *A*: HDLEC were subjected to the same shear protocol using Ca^2+^-containing physiologic saline solution (PSS; 2 mM) showed 1st and 2nd peaks that were significantly different from their baseline. The 2nd peak was also significantly smaller than the 1st peak (means ± SE; *n* = 9). *B*: exposing HDLEC to the same shear protocol using Ca^2+^-free PSS (0 mM) showed an increase for the 1st stimulus but the 2nd stimulus failed to increase the [Ca^2+^]_i_ signal to levels significantly higher than the baseline. The 1st peak was also significantly higher than the 2nd peak (means ± SE; *n* = 9). *C*: comparing the results from Ca^2+^-containing PSS with Ca^2+^-free PSS showed that both 1st and 2nd peaks were larger in Ca^2+^-containing PSS than in Ca^2+^-free PSS. The peaks for *C* are calculated in each experiment individually and then are averaged over the number of experiments. *D*: comparisons among Ca^2+^-containing PSS (blue), Ca^2+^-free PSS (green), and the calculated difference between these 2 signals (red) that represents the contribution of extracellular calcium entry indicated that the peak of external calcium happened 1.5 min later than the peak of Ca^2+^-free PSS (*P < 0.05; ns: nonsignificant).

To investigate the effects of extracellular calcium concentration on shear-induced [Ca^2+^]_i_ signal, the cells were sheared using PSS solutions with either 0 or 2 mM Ca^2+^ as described in methods (Fig. 3, *A* and *B*). Shearing in 0 mM Ca^2+^ attenuated the first peak and completely abolished the second peak compared with its own baseline, indicating a contribution of extracellular calcium entry in the shear-induced calcium response. The height of the first peak in Ca^2+^-containing PSS (0.33 ratio, baseline subtracted) was significantly higher than the first response in Ca^2+^-free PSS (0.19 ratio) when 10 dyn/cm^2^ shear was applied to the HDLEC (Fig. 3*C*). This indicates that the calcium signal is dependent on both intracellular and extracellular sources (Fig. 3*D*). To have a better representation of the extracellular calcium entry contribution, the difference between Ca^2+^-containing signal and Ca^2+^-free signal was calculated (Fig. 3*D*). The extracellular calcium entry appears to reach a peak 1.5 min after the peak from Ca^2+^-free PSS (representing only the intracellular calcium release). Additionally, the second peak from Ca^2+^-free PSS was not significantly different from the baseline signal before the stimulus (Fig. 3*B*).

**Fig. 4.**
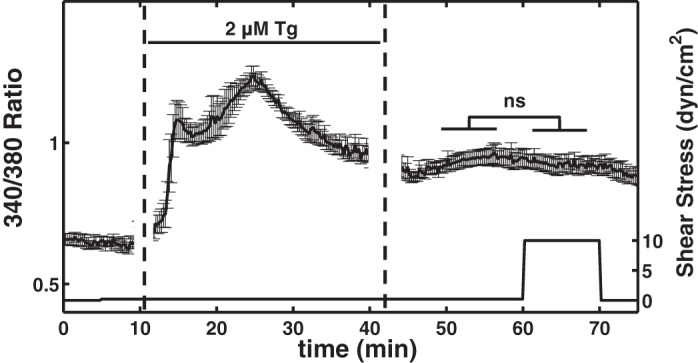
Effect of thapsigargin (Tg) on shear-mediated calcium changes. After the baseline signal was read in the 1st 5 min, HDLEC were perfused with a small flow of DMEM/F-12 (∼0.2 dyn/cm^2^) for another 5 min. The media were then switched to DMEM/F-12 with 2 µM Tg with same slow perfusion rate. The HDLEC exhibited 2 consecutive transient peaks and approached toward a new stable elevated level. At this point the media were switched back to normal DMEM/F-12 when the new baseline was recorded and cells were subjected to 10 dyn/cm^2^ shear stress for 10 min. Shear stress did not change the signal significantly in this experiment (means ± SE; *n* = 6, ns: nonsignificant).

Since the calcium signal was not completely inhibited with the removal of calcium from extracellular buffer, experiments were performed to determine the role of ER calcium stores in the shear-mediated [Ca^2+^]_i_ transients using available calcium ER pump blockers. Thapsigargin (Tg) is known to irreversibly block the sarco/endoplasmic reticulum Ca^2+^-ATPase (SERCA) pumps responsible for calcium reuptake from the cytoplasm back into the ER, maintaining low basal cytoplasmic calcium concentration. Upon treatment with 2 μM Tg, [Ca^2+^]_i_ increased substantially, reaching an initial peak in 4.9 min followed by a slight dip in calcium before achieving a second higher peak after 8.2 min. This signal then decayed from the highest peak levels to a stable level (0.99 ± 0.04) that was significantly higher than the initial basal level (0.64 ± 0.02). These Tg-pretreated HDLEC were then subjected to 10 dyn/cm^2^ but this did not produce a significant further change in [Ca^2+^]_i_ (Fig. 4). The complete disappearance of the shear response (to 10 dyn/cm^2^ shear stress) after SERCA blockade suggests an important role for ER calcium release in the generation of the shear-induced calcium response.

**Fig. 5.**
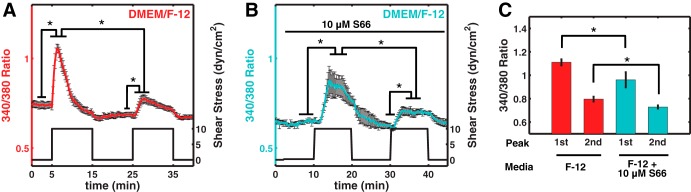
Effect of a calcium release-activated calcium (CRAC) channel blocker S66 on [Ca^2+^]_i_ dynamics to shear. *A*: shear-mediated calcium transient under 10 dyn/cm^2^ using DMEM/F-12 is shown here (similar to Fig. 2*C*; means ± SE; *n* = 9). *B*: after the baseline was recorded for 2 min, HDLEC were perfused slowly (∼0.2 dyn/cm^2^) with 10 µM S66 in DMEM/F-12 for 8 min. Then a similar shear stress waveform to previous experiments was used to investigate the effect of blocking CRAC channels flow-induced calcium signals (means ± SE; *n* = 5). The 1st and 2nd peaks were both higher than their baselines. The 2nd peak was also significantly smaller than the 1st peak. *C*: blocking CRAC channels by S66 resulted in smaller 1st and 2nd peaks compared with control experiments with DMEM/F-12 under 10 dyn/cm^2^. The peaks for *C* are calculated in each experiment individually and then are averaged over the number of experiments (*P < 0.05).

The specific role of calcium release-activated calcium (CRAC) channels in mediating Ca^2+^ entry in response to the ER calcium release was investigated by inhibiting these channels using Syntha66 (also known as S66, a specific CRAC channel blocker) (14, 29, 36), thereby revealing their contribution to the shear-mediated [Ca^2+^]_i_ signal (Fig. 5, *A* and *B*). Blockade of CRAC channels decreased the height of both the first (1.02 ± 0.03) and second peaks (0.73 ± 0.01) relative to their untreated controls (Fig. 5*C*). Moreover, S66 treatment often produced a delay of about 2–5 min in the shear-induced calcium response. Specifically, S66 increased the time to reach the first peak to 5.2 ± 0.4 min (2- to 5-min delay + an increase with a rate of 0.165 ratio/min; Table 1) compared with 1.8 ± 0.1 min without CRAC channel blockade (Fig. 5, *A* and *B*).

**Table 1.** Shear stress level, extracellular calcium concentration, and curve-fit parameters for the experiments

			First Peak		Second Peak	
Media	Shear Stress, dyn/cm^2^	Media Calcium Concentration, mM	Upstroke slope–*a*_1_, ratio/min	Downstroke time const.–*b*_2_, min	Upstroke slope–*a*_1_, ratio/min	Downstroke time const.–b_2_, min
DMEM/F-12	1	1.05	0.058	4.6	0.023	10.2
DMEM/F-12	3	1.05	0.192	3.9	0.063	16.1
DMEM/F-12	10	1.05	0.302	3.0	0.057	11.5
DMEM/F-12 (30 min between 2 stimuli)	10	1.05	0.258	2.7	0.123	5.1
DMEM/F-12 + 10 μM S66	10	1.05	0.165	5.0	0.053	28.9
PSS	10	2.00	0.163	2.3	0.083	2.6
Ca-free PSS	10	0.00	0.167	2.6	0.024	3.5

PSS, physiologic saline solution.

**Fig. 6.**
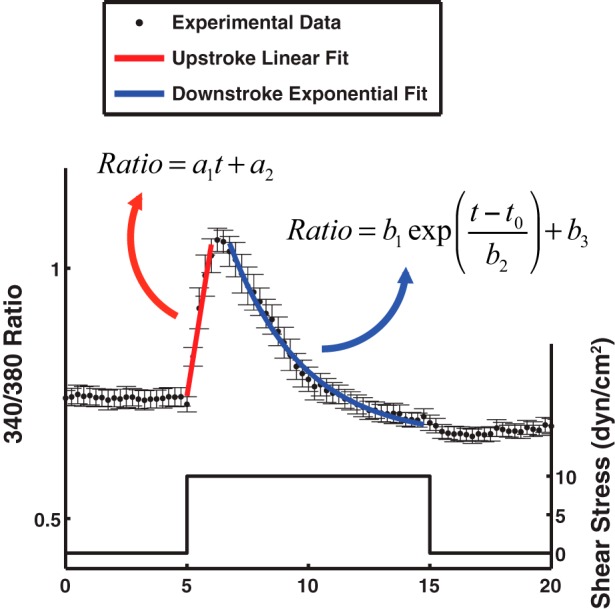
Curve-fit to the upstroke and downstroke of the calcium spike. The 1st peak of the calcium response to 10 dyn/cm^2^ shear stress is shown with filled circles (●; means ± SE; *n* = 9). A sample linear fit of the form ratio = *a*_1_ × *t* + *a*_2_ to the upstroke (the red line) and a sample exponential fit of the form ratio = *b*_1_ × exp[(*t* − *t*_0_)/*b*_2_] + *b*_3_ to the downstroke (the blue curve) are shown in this figure. *a*_1_ and *b*_2_ are the main parameters that are reported in the Table 1.

The calcium responses to shear were fit to mathematical function to extract parameters for subsequent modeling efforts (Fig. 6). The [Ca^2+^]_i_ increased rapidly to reach a peak value, which was approximated using a linear function in the form of ratio = *a*_1_ × *t* + *a*_2_ (Fig. 6, red line). The gradual drop in signal suggested an exponential fit {ratio = *b*_1_ × exp[(*t* − *t*_0_)/*b*_2_] + *b*_3_} as an adequate approximation for this part of the response (Fig. 6, blue curve). Among the curve-fitting parameters, the upstroke slope showing the [Ca^2+^]_i_ increase rate (*a*_1_) and the downstroke time constant (*b*_2_) are the most important parameters and are reported for the various cases tested in this study for the first and second peaks (Table 1). When the shear stress increased from 1 to 10 dyn/cm^2^ in the absence of inhibitors, the upstroke slope increased sixfold for the first peak suggesting a faster increase in the signal, and the downstroke time constant decreased from 4.6 to 3.0 min meaning that the signal dropped faster when the shear stress was higher (Table 1).

## DISCUSSION AND CONCLUSION

**Fig. 7.**
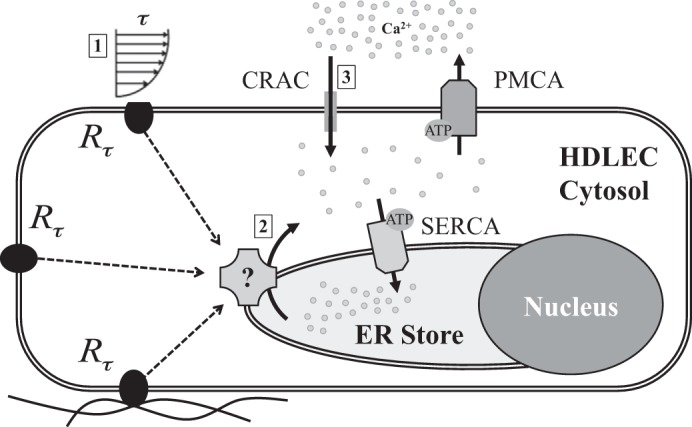
Schematic of shear-mediated calcium dynamics in HDLEC. The results of this work suggest that following shear stress mechanosensing (*R*_τ_) in HDLEC (*1*), the intracellular calcium increases due to endoplasmic reticulum (ER) store calcium release (*2*). The increase in [Ca^2+^]_i_ initiates Ca^2+^ entry to the cell through CRAC channels (*3*). Plasma membrane Ca^2+^ ATPase (PMCA) and the sarco/endoplasmic reticulum Ca^2+^-ATPase (SERCA) pumps are constantly pumping the cytosolic Ca^2+^ to the extracellular space and ER store, respectively.

Our findings demonstrate that HDLEC respond to physiologically relevant shear stress with a transient increase in [Ca^2+^]_i_ arising from a combination of release from intracellular stores and entry of extracellular calcium (Fig. 7). With the onset of a constant shear stress, [Ca^2+^]_i_ increased quickly and peaked within minutes (usually 100–200 s). This was followed by a gradual decay to levels often below the initial basal concentrations. This calcium response is quite different from that observed with blood EC, where the signal increases in a shorter time and sometimes a sustained elevation in cytosolic calcium is observed. However, the shear-dependent response of calcium in blood EC has been shown to be dependent on numerous factors in addition to shear (Table 2) (17, 31, 55). Because this is the first study of this nature done in lymphatic EC, it is difficult at this time to know if these factors play a significant role in the shear-sensitive calcium response we observed. Further studies need to be done to carefully evaluate these factors and the apparent differences in blood and lymphatic EC response to shear. Dull and Davies (17) observed a shear-induced [Ca^2+^]_i_ spike that peaked at 134 nM in 6–10 s for shear stresses under 6.3 dyn/cm^2^ in bovine aortic endothelial cells (BAEC) that was only observed when ATP was present in the media. The current study uses DMEM/F-12 lacking ATP, yet a [Ca^2+^]_i_ peak is present when stimulated by shear stress. Additionally, the shear-induced peak time for BAEC with ATP-containing media is much shorter than shear-induced HDLEC with ATP-free media (6–10 vs. 100–200 s). Liu et al. (31) also measured [Ca^2+^]_i_ in BAEC using fluorescence resonance energy transfer (FRET) biosensors under shear stress and observed a doubling in FRET signal in ∼10 s that was sustained during the first 300 s. This signal dropped to ∼40% after 1,000 s. The time to peak is shorter than the present study (10 vs. 100–200 s), and the rapid decay that was observed in HDLEC in the present study is different from sustained increase in [Ca^2+^]_i_ measured in BAEC by Liu et al. In another study, Shen et al. (42) measured [Ca^2+^]_i_ in BAEC under shear stress and observed a peak of ∼137 nM in 15–40 s under shear stresses higher than 4 dyn/cm^2^, which declined to basal levels in 40–80 s. Similar to other studies on BAEC, this peak time is shorter than that observed using HDLEC, while the peak values are similar. Schwarz et al. (41) applied shear stress for a period of 60 s to individual human umbilical vein EC (HUVEC) and measured [Ca^2+^]_i_. After an initial increase in signal, they either observed an instantaneous decay of the signal at the offset of shear stress, or the signal continued to increase after shear stopped and reached a peak followed by a decay back to baseline. For a shear of 25 dyn/cm^2^, [Ca^2+^]_i_ peaked at 530 nM with time constant of exponential decay reported to be 84 s compared with the HDLEC response to 10 dyn/cm^2^ in this study that peaked at 126 nM with a time constant of 180 s. The time constant measured by Schwarz et al. is during the period that the shear was stopped while in this study the time constant was measured while shear was still applied. While the actual peak time is not reported by this group, the fact that [Ca^2+^]_i_ signal did not reach a peak during first 60 s suggests HUVEC have a more similar peak time compared with HDLEC than do BAEC.

**Table 2.** Summary of shear-mediated calcium dynamics data available in the literature

		Peak Response			Experimental Condition		Culture Condition				
Article	Cell Type	Peak signal	Peak Time	Shear Stress Level	Technique	Exp. buffer	Media	ECM	Seeding density	Passage #	Time between seeding and exp.
Current study	HDLEC	126.2 ± 5.8 nM	100–200 s	10 dyn/cm^2^	2 μM fura-2 AM	DMEM/F-12	MV2	2% gelatin	50%	<6	3 days
Dull and Davies (17)	BAEC	134 nM (avg)	6–10 s	6.3 dyn/cm^2^	5 μM fura-2 AM	DPBS with 1.5 mM Ca^2+^ + 2 μM ATP	DMEM + HEPES + 10% calf serum	0.1% gelatin			
Shen et al. (42)	BAEC	137 nM (avg)	15–40 s	4 dyn/cm^2^	2 μM fura-2 AM	HBSS with 1.5 mM Ca^2+^	DMEM +10% calf serum			5–30	After confluence
Liu et al. (31)	BAEC	2-fold	10 s	65 dyn/cm^2^	FRET biosensor	DMEM +10% FBS	DMEM +10% FBS				
Schwarz et al. (41)	HUVEC	587 ± 88 nM	∼60 s	∼25 dyn/cm^2^	2 μM fura-2 AM	Krebs solution with 1.5 mM Ca^2+^	Medium 199 + 1 mg/ml ATP +10% human serum	Gelatin		<2	2–4 days

DMEM, Dulbecco's modified Eagle's medium; MV2, endothelial cell growth medium MV2; DPBS, Dulbecco's phosphate-buffered saline; HBSS, Hanks' balanced salt solution; FRET, fluorescence resonance energy transfer; FBS, fetal bovine serum; HDLEC, human dermal lymphatic endothelial cells; BAEC, bovine aortic endothelial cells; HUVEC, human umbilical vein endothelial cells; ECM, extracellular matrix.

The shear-induced rate of increase of [Ca^2+^]_i_ was more sensitive to shear magnitude in the low shears (0–1 dyn/cm^2^) compared with the high shear ranges (3–10 dyn/cm^2^; Table 1). This suggests either a higher mechanosensitivity of HDLEC to shear stress in low shear ranges or activation of a strong calcium extrusion mechanism simultaneously that requires higher shears to be fully activated. The shear stress environment to which LEC and blood EC are subjected are quite different. Local shear stress in the lymphatics is dependent on the type of lymphatic vessel involved and the conditions to which that lymphatic vessel is currently exposed. Furthermore, lymph flow in a given lymphangion is highly complex and dynamic due to variable levels of lymph formation upstream coupled with the periodic contraction/relaxation of lymphangions. In rat mesenteric prenodal collecting lymphatics, the resulting wall shear stresses vary dynamically from slightly negative to ∼12 dyn/cm^2^ in straight segments away from the valves in a matter of a few seconds (16). In addition, because of the heterogeneous shape of these vessels, shears vary dramatically along the lymphangion and are up to two to three times higher on the valve leaflets compared with the downstream straight segments (54). Furthermore, shear stresses can increase 10 times with the increases in lymph formation seen during edemagenic fluid balance shifts (38). To the degree that it is relevant to describe flow in a collecting lymphatic with a time-average value, under basal physiological conditions shear stress was ∼0.64 dyn/cm^2^ in the straight lymphatic segments (16). However, it is important to note that LEC are only instantaneously and irregularly subjected to this level of shear stress as flow accelerates or decelerates. Shear stresses in precollectors and lymphatic capillaries are even lower [∼0.001 in mouse tail capillaries (5) and ∼0.003 dyn/cm^2^ in human skin capillaries (18)]. Given the dynamics of this vascular system, it is reasonable to assume LEC are sensitive to transients in shear stress. The results of this study support that hypothesis and further suggest that HDLEC may respond to smaller transient increases in shear stress than blood EC. Our results indicate that the shear-mediated [Ca^2+^]_i_ response is sensitive to shear magnitude between 0 and 3 dyn/cm^2^ (Fig. 2*D*). With increases in shears to levels above that (10 dyn/cm^2^), the response does not appear to be much different from that seen at 3 dyn/cm^2^. Comparing our data with shear-mediated [Ca^2+^]_i_ responses in BAEC supports this hypothesis. Specifically, Liu et al. (31) showed a relatively linear increase in the shear-mediated [Ca^2+^]_i_ response in BAEC in the shear stress range of 15 to 65 dyn/cm^2^ suggesting BAEC are sensitive to a higher range of shear magnitudes compared with HDLEC. These data from BAEC contradict the study by Shen et al. (42) that reported a signal plateau at 4 dyn/cm^2^ for BAEC. Schwarz et al. (41) applied shear stresses of up to 52 dyn/cm^2^ to HUVEC and observed that the response to shear reached a plateau at ∼40 dyn/cm^2^, which is still higher than that observed in HDLEC in this study. Kawai et al. (24) reported a significant upregulation in eNOS (a molecular signal in the NO pathway downstream of [Ca^2+^]_i_) after treatment of collecting LEC with shear stresses between 0.5 and 1 dyn/cm^2^ for 2 h, indicating a sensitivity to low levels of shear stress. Furthermore, these patterns of shear-sensitive calcium responses and eNOS expression in cultured LEC in general match the patterns of eNOS expression and NO production we have observed experimentally in rat mesenteric collecting lymphatics in situ (9, 10) and predicted computationally (54).

This study also attempts to distinguish between the role of extracellular calcium entry vs. ER calcium store release in the resulting [Ca^2+^]_i_ signal. Reduction of the shear-induced calcium response as a result of the calcium removal from the external buffer (cf. Fig. 3) suggests that the observed calcium rise was in part due to extracellular calcium influx (40% of the first peak height). A similar conclusion was made by Shen et al. (42) when they used EGTA to chelate Ca^2+^ ions in the buffer before applying shear stress on BAEC. In contrast, Schwarz et al. (41) observed that the [Ca^2+^]_i_ signal was completely abolished using Ca^2+^-free buffer. Our results are consistent with the hypothesis that the [Ca^2+^]_i_ signal generated in HDLEC subjected to shear in the absence of external calcium comes from intracellular calcium stores, most likely from ER calcium release. We kept the pretreatment time in Ca^2+^-free solution to a minimum (2–3 min) in these experiments to minimize the potential loss of intracellular calcium stores that occurs when cells are maintained in a calcium-free environment. In DMEM/F12 experiments, the magnitude of the second stimulus was significantly lower than the first one for shears higher than 1 dyn/cm^2^. Allowing a longer resting interval between repeated stimuli resulted in a greater response to a second application of shear, which was still significantly lower than the first peak (cf. Fig. 2*E*). This implies that the shear-activated depletion of calcium stores gradually refill between consecutive shear stimuli. A stronger recovery of the second peak in the case with Ca^2+^-containing PSS vs. an nonsignificant recovery in the Ca^2+^-free PSS confirms that external calcium is necessary for the full restoration of the intracellular ER calcium stores ([Ca^2+^]_ER_). When [Ca^2+^]_i_ increases, the plasma membrane Ca^2+^ ATPase (PMCA) pumps cytosolic Ca^2+^ to the extracellular space and SERCA pumps return cytosolic Ca^2+^ back into the ER to reinstate a low cytoplasmic calcium level. The lack of a significant second peak generated in the absence of extracellular Ca^2+^ indicates that the SERCA pumps may not have effectively restored [Ca^2+^]_ER_ after the shear-stimulated calcium release due to limited [Ca^2+^]_i_. Thus when extracellular calcium is present, recovery of the second peak [Ca^2+^]_i_ response can be achieved by increasing the time between two stimuli and hence providing more time to restore the initial [Ca^2+^]_ER_.

Using BAEC, Liu et al. (31) concluded that the first phase of the shear induced calcium response is caused by extracellular calcium entry and the prolonged increase is from intracellular sources (31). Our data in HDLEC show significant differences between BAEC and HDLEC in this respect. In HDLEC, neither the timing nor the slope of the initial rise in the signal is significantly altered by removal of the extracellular calcium (cf. Fig. 3 and Table 1), indicating that the initial phase of the [Ca^2+^]_i_ rise after shear is mainly due to the release of calcium from the ER. The relative contribution of the intracellular and extracellular calcium to the shear-dependent [Ca^2+^]_i_ rise is indicated in Fig. 3*D* and suggests that extracellular calcium influx increases the peak magnitude and duration. The elevation in calcium is then followed by a reduction to levels that were often lower than the initial baseline. This unique feature has not been observed in the shear mediated calcium changes in blood EC and suggests that cytoplasmic calcium removal systems in HDLEC have been activated by shear. Moreover, the decay time constant (*b*_2_ in Table 1) appears to be dependent on the shear stress magnitude and decreases as the magnitude increases, thus supporting the shear-activated Ca^2+^ extrusion hypothesis. Mathematical modeling of agonist-induced [Ca^2+^]_i_ dynamics in arterial EC has been developed that includes contributions from channels and pumps on the plasmalemmal and ER membrane (25, 44, 53). The effects of shear stress are also included in some models describing [Ca^2+^]_i_ dynamics in arterial EC (53). However, these models are not able to capture the reduction of calcium below basal levels we observed in HDLEC. We suspect that this particular characteristic of the [Ca^2+^]_i_ response in HDLEC is caused by the activation of extracellular and/or intracellular calcium pumps that are either not present or do not have significant effects in blood EC. Further studies are needed to investigate this important and unique shear-dependent characteristic of HDLEC and to identify the responsible calcium extrusion mechanisms.

Tg (a SERCA inhibitor) administration completely eliminated the shear-induced response, suggesting a critical role for SERCA pumps in the shear-induced calcium response. These data, in combination with the experiments on the effect of extracellular calcium, suggest that extracellular calcium entry is significantly activated only when the store release process is initiated. Blocking SERCA pumps eventually leads to depletion of ER stores and in turn activates the CRAC channels resulting in the elevation of the calcium signal while under no shear. However, in addition to the depletion of ER stores, the rise in the cytosolic calcium caused by Tg may have also contributed to the loss of the shear-mediated signal. Although Tg is widely used in calcium studies as the first candidate for SERCA inhibition, it may affect other cellular pathways as well. To further delineate the plasma membrane calcium channels involved in the extracellular calcium influx, we investigated the role of CRAC channels using the specific inhibitor S66. S66 blockade of CRAC channels reduced the magnitude of the shear-induced calcium rise, although inhibition of the extracellular calcium entry through CRAC channels did not diminish the calcium rise to the same degree as exposure to calcium free media. The blockade of CRAC channels reduced both the first and second peaks of [Ca^2+^]_i_ for 10 dyn/cm^2^ shear relative to the control and caused a significant delay of 2–5 min in the initiation of the [Ca^2+^]_i_ response to shear. The mechanisms by which the CRAC channel inhibition by S66 delays the onset of the initial calcium rise to shear are not clear and have not been reported before in other EC lines. Because removing extracellular Ca^2+^ did not lead to a significant delay of shear-induced [Ca^2+^]_i_ signal, it is possible that the delay caused by S66 incubation is due to some unknown effects of S66. It is also possible that preincubation of HDLEC with S66 reduces store content by blocking Ca^2+^ entry through CRAC channels under the basal status. Thus it appears that CRAC channels play a significant role in the rise of [Ca^2+^]_i_ induced by shear in HDLEC and other calcium-permeable channels in the plasmalemmal may also be involved in this process. Further studies on the identities and specific roles of these channels in HDLEC remain to be elucidated.

Although there is a wide range of temporal and spatial variance in shear stress magnitude applied to LEC in different vessels throughout the lymphatics, the step changes in shear stress used here serve to illustrate the basic dynamics behind calcium responses to temporal changes in shear stress. Given the variety of shear stresses to which LEC are exposed in vivo, it makes sense to initially “standardize” the shear stress waveform for these kinds of experiments. Future experiments should investigate responses to specific in vivo shear stress waveforms, but these would not be expected to significantly alter the conclusions drawn from this study. The images in this study were captured every 15 s, which means the temporal resolution of the results in this study is limited by this time step. Although in vitro studies provide good control over the mechanical forces applied on the LEC, they inherently lack the detailed microenvironmental features present in vivo such as the stiffness and complex composition of extracellular matrix and lymph composition that may play important roles in LEC mechanosensing. The extent to which these differences can affect the results needs to be addressed in future studies on LEC flow responses. Additionally, the LEC mechanosensors and transduction pathways are yet to be elucidated. The role of ATP-binding purinergic P2X/2Y receptors has been emphasized by several studies (17, 55) in arterial EC; however, there exist several other candidates such as glycocalyx complexes on the plasma membrane (46), primary cilia (35), and numerous ion channels (13) that can mediate this signal. Although the baseline media (DMEM/F-12) used in this study was ATP-free, endogenous ATP release could still potentially stimulate P2X/2Y receptors. This work suggests that at least one HDLEC mechanosensor initiates a pathway resulting in ER store Ca^2+^ release followed by extracellular Ca^2+^ entry; however, further experiments are necessary to identify the details of this pathway in HDLEC. Commercial HDLEC lines are isolated in a manner in which we know they are lymphatic in origin although we cannot determine exactly which type of lymphatic vessels they represent. The HDLEC line used in this study contains a mixture of EC from collecting and capillary lymphatics in the human foreskin that is expected to exhibit heterogeneity in their response. These HDLEC have been used extensively by scientists in the field and thus represent a good example to allow comparisons with others work on lymphatic endothelium. Lastly, it is also not clear how cultured HDLEC may change in vitro under typical culture conditions, although this general approach has been broadly and effectively utilized to study BAEC.

In summary, because of well-defined sensitivities of lymphatic vessels in vivo and in vitro to flow/shear in terms of modulating lymphatic tone, lymph pumping, and nitric oxide production, we evaluated the shear-sensitive changes in HDLEC cytoplasmic calcium. We measured [Ca^2+^]_i_ dynamics in cultured HDLEC in response to step changes in physiologically relevant shear stress and showed that the magnitude of the calcium rise is dependent on the magnitude of shear stress. Furthermore, we have identified specific calcium sources that contributed to this signal. We demonstrated that the intracellular calcium store release was mainly responsible for the peak observed after the shear started, whereas extracellular calcium entry contributed to the magnitude and duration of the calcium signal. Furthermore, shear was not able to initiate the spike when the ER stores had been depleted. Further investigations are necessary to refine the mechanotransduction signaling pathway responsible for [Ca^2+^]_i_ sensitivity to shear in HDLEC.

## GRANTS

We acknowledge National Heart, Lung, and Blood Institute Grants R01-HL-094269, HL-096552, and HL-070308 and American Heart Association 13SDG17200006 for funding this project. J. E. Moore wishes to acknowledge the support of The Royal Society, The Royal Academy of Engineering, and The Sir Leon Bagrit Trust.

## DISCLOSURES

No conflicts of interest, financial or otherwise, are declared by the author(s).

## AUTHOR CONTRIBUTIONS

Author contributions: M.J., R.R.K., S.L.Z., D.C.Z., and J.E.M. conception and design of research; M.J. and W.E.C. performed experiments; M.J., W.E.C., R.R.K., S.L.Z., D.C.Z., and J.E.M. analyzed data; M.J., W.E.C., R.R.K., S.L.Z., D.C.Z., and J.E.M. interpreted results of experiments; M.J. prepared figures; M.J. drafted manuscript; M.J., W.E.C., R.R.K., S.L.Z., D.C.Z., and J.E.M. edited and revised manuscript; D.C.Z. and J.E.M. approved final version of manuscript.
